# Violacein Induces Death of Resistant Leukaemia Cells via Kinome Reprogramming, Endoplasmic Reticulum Stress and Golgi Apparatus Collapse

**DOI:** 10.1371/journal.pone.0045362

**Published:** 2012-10-11

**Authors:** Karla C. S. Queiroz, Renato Milani, Roberta R. Ruela-de-Sousa, Gwenny M. Fuhler, Giselle Z. Justo, Willian F. Zambuzzi, Nelson Duran, Sander H. Diks, C. Arnold Spek, Carmen V. Ferreira, Maikel P. Peppelenbosch

**Affiliations:** 1 Center for Experimental and Molecular Medicine, Academic Medical Center, University of Amsterdam, Amsterdam, The Netherlands; 2 Department of Biochemistry, Institute of Biology, University of Campinas, Brazil (UNICAMP), Campinas, São Paulo, Brazil; 3 Department of Gastroenterology and Hepatology, Erasmus MC-University Medical Center, Rotterdam, The Netherlands; 4 Department of Cell Biology and Department of Biochemistry, Federal University of São Paulo (UNIFESP), São Paulo, São Paulo, Brazil; 5 Multidisciplinary Lab in Dental Research, Heath Sciences School, University of Grande Rio (UNIGRANRIO), Rio de Janeiro, Brazil; 6 Biotechnology Lab, Bioengineering Sector, National Institute of Metrology, Quality and Technology (Inmetro), Duque de Caxias, Rio de Janeiro, Brazil; 7 Biological Chemistry Laboratory, Institute of Chemistry, University of Campinas (UNICAMP), Campinas, São Paulo, Brazil; 8 Beatrix Children's Hospital, Department of Pediatric Oncology, University Medical Center Groningen, University of Groningen, Groningen, The Netherlands; Universidade Federal do Rio de Janeiro, Brazil

## Abstract

It is now generally recognised that different modes of programmed cell death (PCD) are intimately linked to the cancerous process. However, the mechanism of PCD involved in cancer chemoprevention is much less clear and may be different between types of chemopreventive agents and tumour cell types involved. Therefore, from a pharmacological view, it is crucial during the earlier steps of drug development to define the cellular specificity of the candidate as well as its capacity to bypass dysfunctional tumoral signalling pathways providing insensitivity to death stimuli. Studying the cytotoxic effects of violacein, an antibiotic dihydro-indolone synthesised by an Amazon river *Chromobacterium*, we observed that death induced in CD34^+^/c-Kit^+^/P-glycoprotein^+^/MRP1^+^ TF1 leukaemia progenitor cells is not mediated by apoptosis and/or autophagy, since biomarkers of both types of cell death were not significantly affected by this compound. To clarify the working mechanism of violacein, we performed kinome profiling using peptide arrays to yield comprehensive descriptions of cellular kinase activities. Pro-death activity of violacein is actually carried out by inhibition of calpain and DAPK1 and activation of PKA, AKT and PDK, followed by structural changes caused by endoplasmic reticulum stress and Golgi apparatus collapse, leading to cellular demise. Our results demonstrate that violacein induces kinome reprogramming, overcoming death signaling dysfunctions of intrinsically resistant human leukaemia cells.

## Introduction

The physiology of metazoan organisms requires mechanisms that direct cellular death in a controlled fashion, a process collectively denominated as programmed cell death (PCD) [1]. During embryology and further development to sexual maturity, various structures like the pro-nephros and mesonephros have only transient functionality and are orderly disposed off when no longer necessary. Analogously, development of the immune system as well as termination of ongoing host-defence responses require the controlled elimination of a large number of effector cells [2]. Furthermore, aberrant compartment size regulation is implicated in many serious pathologies (most dramatically, maybe, in cancer), and prominently involves defects in PCD [3].

Cell-intrinsic programmed suicide has been functionally classified by the Committee on Cell Death 2012 as consisting of, so far, 13 separate molecular modes of cell death: Anoikis, autophagic cell death, caspase-dependent intrinsic apoptosis, caspase-independent intrinsic apoptosis, cornification, entosis, extrinsic apoptosis by death receptors, extrinsic apoptosis by dependence receptors, mitotic catastrophe, necroptosis, netosis, parthanatos and pyroptosis which are distinguished based on biochemical features which are nicely reviewed in [4] and by the Committee on Cell Death 2012 [4], [5].

It is now generally recognised that different modes of PCD are intimately linked to the cancerous process, as apoptotic cell death is an important anti-neoplastic protective mechanism upon improper induction of cellular proliferation, and is also involved in cancer cell clearance by immunosurveillance, chemotherapy or radiotherapy [6]. Autophagy, which counteracts other forms of PCD including apoptosis, may play a role in the escape from chemotherapy by transformed cells [7]. Necroptosis and pyroptosis are important for inducing cell death during inflammatory reactions, thereby preventing inflammation-associated cancer [4]. In addition, chemoprevention of cancer is also thought to be linked to PCD, inducing cellular suicide in pre-neoplastic lesions and thus halting development of full-blown cancer [8]. The mode of PCD involved in cancer chemoprevention is much less clear and may be different between types of chemopreventive agents and tumour cell types involved. Chemoprevention of colorectal cancer by NSAIDS, for instance, is most often linked to a pro-apoptotic response in the transformed compartment [9], but the robust chemoprevention of the same cancer type by the use of statins is linked to necroptosis [10]. Moreover, we have recently presented evidence that chemoprevention induced by apigenin is linked to an autophagic response [7]. Generally speaking however the mode of PCD by many important cancer chemopreventive agents remains poorly characterised, prompting further research in this area.

One such chemopreventive compound is Violacein [3-(1,2-dihydro-5-(5-hydroxy-1H-indol-3-yl)-2-oxo-3H-pyrrol-3-ilydene)-1,3-dihydro-2H-indol-2-one], a purple-colored pigment produced by *Chromobacterium violaceum*, a bacterium present in certain parts of the Amazon river basin in Brazil [11]. Violacein shares an interesting antitumor activity with various other naturally occurring indolones [12]. Violacein has previously been shown to induce apoptosis of HL60 leukaemia cells. Interestingly, TF1 cells which are less differentiated than HL60 seems to be more resistant to violacein-induced apoptosis [13]. However, violacein is still able to induce death in these cells via an alternative mechanism.

In an effort to obtain further insight into the molecular mechanisms of violacein-mediated anti-proliferative responses, we studied the effect of this compound on TF1 leukaemia cells. We observed induction of cellular suicide in these leukaemia cells, and subsequent morphological investigation revealed a mode of cell death involving endoplasmic reticulum and Golgi linearization and ‘horseshoe-shaped’ nucleus. To investigate the mechanism of violacein-induced death in TF1 cells, we performed kinome profiling using peptide arrays to yield comprehensive descriptions of cellular kinase activities. Kinome profiling revealed a calpain based mechanism leading to cellular demise. Thus, our findings demonstrate that this non-canonic mechanism of cell death induced by violacein might explain its strong antileukaemic properties even in cell lines which are less sensitive to classical induction of cell death.

## Materials and Methods

### Reagents

Polyclonal antibodies against ERK1/2 (Thr202/Tyr204), MAPAPK2 (Thr222), p38 (Thr180/Tyr182), mTOR (Ser2448), PKB, phospho-PDK1 (Ser241), phospho-GSK3β (Ser9), LC3B and Beclin1, were purchased from Cell Signaling Technology (Beverly, MA). Secondary anti-rabbit and anti-mouse peroxidase-conjugated antibodies were also obtained from Cell Signaling Technology (Beverly, MA). Antibodies against anti-MAP LC3 and cleaved-PARP and secondary anti-goat antibodies were purchased from Santa Cruz (St. Louis, MO). Anti-Fas receptor (Fas) and anti-Fas ligand (FasL) were from Immunotech (Marseille, France). TNFα was obtained from R&D Systems. Violacein (3-(1,2-dihydro-5-(5-hydroxy-1H-indol-3-yl)-2-oxo-3H-pyrrol-3-ilydene)-1,3-dihydro-2H-indol-2-one) was extracted and purified as previously described [14].

### Cell culture and Treatments

TF1 cell line was purchased from American Type Culture Collection (ATCC, Rockville, MD). Cells were routinely grown in RPMI 1640 culture medium (Gibco) containing 10% fetal bovine serum, 2 mM L-glutamine, 5 ng/ml GM-CSF, 100 units/mL penicillin and 100 µg/mL streptomycin at 37°C in a humidified incubator with 5% CO_2_ in air.

### Cell viability

Cell viability was assessed by trypan blue dye exclusion and MTT reduction assays as previously reported [10], [15].

### Cell Cycle Analysis

TF1 cells were cultured for 24 hours at a density of 2×10^4^ cells/ml in serum free RPMI. After 24 h of serum starvation cells were treated with violacein for 24 h, and subsequently cells were washed with PBS and resuspended in 200 µl of a sodium citrate dihydrate (1 g/L) solution, containing 50 µl Ribonuclease A (10 mg/ml; Fermentas), propidium iodide 0.02 mg/ml (Sigma) and Triton X-100 (Sigma) 0.1%. Next, the cells were incubated in the dark for 60 min at room temperature. The analysis was performed in a FACSCalibur flow cytometer (BD Biosciences, San Jose, CA, USA). The cells were analyzed in low speed and at least 10,000 events were analyzed per sample. The DNA content was evaluated using a FL2 detector in a linear scale. To eliminate cell aggregates, the cell population to be analyzed was selected from a bivariate histogram showing the area (FL2A) versus the width (FL2W) of the signal FL2. The analysis of cell percentage in the different phases of the cell cycle (G0/G1, S, and G2/M) was performed using the ModFit LT software (BD Biosciences, San Jose, CA, USA).

### Western blotting

Cells (3×10^7^) were lysed in 200 µL cell lysis buffer (50 mM Tris [tris(hydroxymethyl)aminomethane]–HCl [pH 7.4], 1% Tween 20, 0.25% sodium deoxycholate, 150 mM NaCl, 1 mM EGTA (ethylene glycol tetraacetic acid), 1 mM *O*-Vanadate, 1 mM NaF, and protease inhibitors [1 µg/mL aprotinin, 10 µg/mL leupeptin, and 1 mM 4-(2-amino-ethyl)-benzolsulfonyl-fluoride-hydrochloride]) for 2 h on ice. Protein extracts were cleared by centrifugation, and the protein concentration was determined using Lowry. An equal volume of 2× sodium dodecyl sulfide (SDS) gel loading buffer (100 mM Tris-HCl [pH 6.8], 200 mM dithiothreitol [DTT], 4% SDS, 0.1% bromophenol blue and 20% glycerol) was added and the samples were boiled for 10 minutes. Cell extracts, corresponding to 3×10^5^ cells, were resolved by SDS-polyacrylamide gel (12%) electrophoresis (PAGE) and transferred to polyvinylidene difluoride (PVDF) membranes. Membranes were blocked in 1% fat-free dried milk or bovine serum albumin (2%) in Tris-buffered saline (TBS)–Tween 20 (0.05%) and incubated overnight at 4°C with appropriate primary antibody at 1∶1000 dilution. After washing in TBS-Tween 20 (0.05%), membranes were incubated with anti-rabbit, anti-goat and anti-mouse horseradish peroxidase-conjugated secondary antibodies at 1∶2000 dilutions (in all Western blotting assays) in blocking buffer for 1 h. Detection was performed by using enhanced chemiluminescence (ECL).

### Transmission electron microscopy

After incubated with violacein, the cells were fixed with 2.0% phosphate-buffered glutaraldehyde. The cells were then postfixed in 1% phosphate-buffered OsO_4_, and embedded in Spurr's resin. Thin sections (0.12 µm) were cut, double stained with UO_2_(CH_3_COO)_2_ (uranyl acetate) and Pb_3_C_12_H_10_O_14_ (lead citrate), and visualized with a Philips TECNA10 transmission electron microscope (TEM). Fifty cells from randomly chosen TEM fields were analyzed for each treatment or control [16].

### Kinomic array

Kinome arrays were performed essentially as described before. [17]–[19]. In short, cells were washed in PBS and lysed in a non-denaturing complete lysis buffer. The peptide arrays (Pepscan, Lelystad, The Netherlands), containing up to 1024 different kinase substrates in triplicate, were incubated with the cell lysates for 2 h in a humidified incubator at 37°C. Subsequently, the arrays were washed in 2 M NaCl, 1% Triton-X-100, PBS, 0.1% Tween and water; thereafter slides were exposed to a phospho-imaging screen for 24–72 h and scanned on a phospho-imager (Fuji, Stanford, USA). The level of incorporated radioactivity, which reflects the extent of phosphorylation, was quantified with specific array software (EisenLab ScanAlyze, version 2.50). Datasets from chips were then analyzed statistically using PepMatrix, as described by Milani et al. 2010. Basically, spot replications were scrutinized for consistency using two indexes: one being the standard deviation∶average (SD/A) ratio and the other being the ratio between the average and the median (A/M) of all three replications for each chip. Parameters applied to the indexes were an SD/A<50% and 80%<A/M<120%. The fold change in phosphorylation between control and treated cells was assessed using Student's t-test, with P<0.05 indicating significance. Distribution of shared events in TF1 cells in response to violacein treatment was visualized using Venny (Oliveros, J.C. (2007) VENNY http://bioinfogp.cnb.csic.es/tools/venny/index.html) and heat map was constructed in R 2.12.0 (R Foundation for Statistical Computing, Vienna, Austria) using heatmap.2.

### Statistical evaluation

The Western blots represent 3 independent experiments. Cell viability data were expressed as the means ± standard deviation of 3 independent experiments carried out in triplicates. Data from each assay were analyzed statistically by ANOVA. Multiple comparisons among group mean differences were checked with the Tukey test. Differences were considered significant when the p value was less than 0.05.

## Results

### Violacein displays antiproliferative action on TF1 leukaemia cells

**Figure 1:**
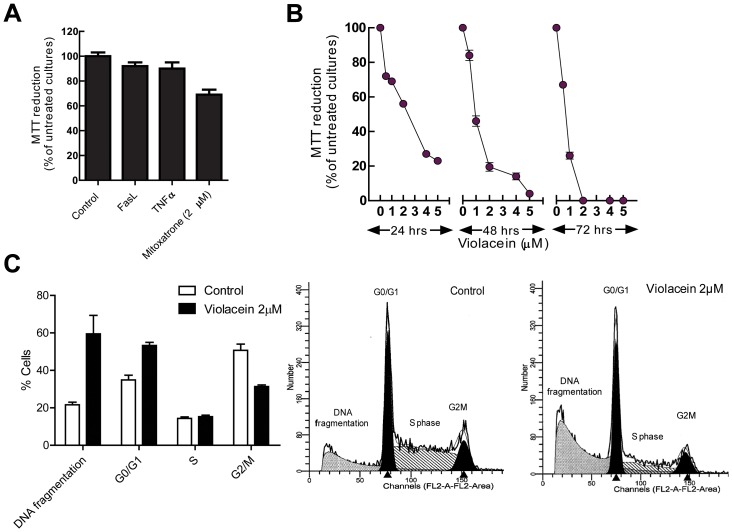
Induction of cell death by violacein in intrinsically resistant TF1 myeloerythroid leukaemia cells. (A) TF1 cells are unusually resistant with respect to PCD. Cells were exposed for 24 hrs to FasL, TNFα and mitoxantrone. (B) TF1 cells were exposed to various concentrations of violacein (X-axis) in the presence of 10% FCS and analysed for cellular survival (as assayed by the capacity of cellular cultures to reduce MTT; Y-axis) 24 hrs, 48 hrs and 72 hrs later as indicated. (C) Analysis of cellular DNA content by FACS shows that violacein-induced TF cell death is accompanied by breakdown of the genome. Each value represents the mean ± SEM of three independent experiments.

CD34^+^/c-Kit^+^/P-glycoprotein^+^/MRP1^+^ TF1 leukaemia progenitor cells have been reported to be unusually resistant against PCD [20]. In apparent agreement, we observed that neither stimulation with high concentrations of Fas ligand, tumour necrosis factor (TNF)α or strong chemotherapeutic agents like mitoxatrone for 24 hours affected TF1 survival to a large degree as assessed by MTT reduction (**figure 1a**). Importantly, however, these cells turned out to be sensitive to violacein, with clear effects on MTT reduction already being evident in the low µM range after 24 h and 2 µM violacein was incompatible with TF1 cell survival after 72 h (**figure 1b**). Subsequent analysis showed that the cell death by violacein is not accompanied by loss of trypan blue exclusion or relevant numbers of annexin V/PI-positive cells (control 9.5%±0.76 and Violacein 2 µM 15.6%±0.5) (not shown); suggesting that the effect of violacein is not due to direct toxic necrosis or classical apoptosis but to a specific cell death programme. In addition, analysis of TF1 cell DNA content shows that violacein-dependent cell death was accompanied by destruction of the genome (**figure 1c**). Previously, we have shown that violacein induces apoptosis of HL60 leukaemia [23]. However, due to the fact that TF1 cells are quite resistant to traditional cell death inductors, such as mitoxantrone and TNFα and violacein managed to overcome this phenotype, we next examined the molecular mechanism by which violacein promotes TF1 cell death.

### Violacein does not induce cellular suicide through the canonical PCD modes

**Figure 2:**
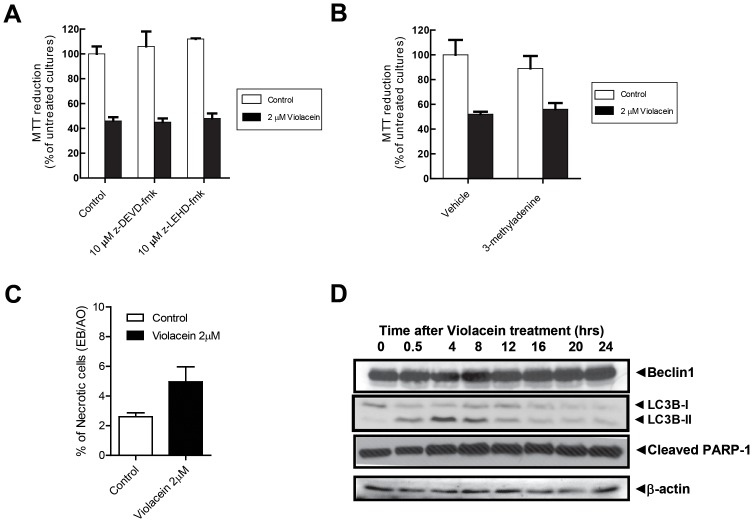
Induction of cell death by violacein does not progress through canonical modes of programmed cell death. (A) Cell death induced in TF1 cells by a 24 hrs treatment with 2 µM of violacein is not sensitive to inhibitors of pro-apoptotic caspases. (B) The autophagosome inhibitor 3-methyladenine does not impair violacein-induced cellular suicide in TF1 cells. (C) Apparent absence of a necrotic effect of violacein in TF1 cells, as assayed by the ethidium bromide/acridine orange assay. (D) Western blot analysis of beclin-1 and autophagy-specific LC3B isoform levels excludes a major role of autophagy in violacein effects in TF1 cells. Nevertheless, violacein clearly increases levels of cleaved PARP and thus cell death induction by violacein represents a mode of PCD.

PCD can progress through different types of molecular system which will ultimately cause cell death [4]. However, a dominant PCD mechanism was not observed in TF1 cells treated with violacein. Caspase-dependent apoptosis is excluded since inhibitors of pro-apoptotic caspases did not affect violacein-dependent cell death (**figure 2a**). Also, the known class III PI3K inhibitor, 3-methyladenine (3-MA, 5 mM), which inhibits the formation of autophagosomes [21], has no effect on violacein-induced cell death (**figure 2b**). Furthermore, the apparent absence of a necrotic effect of violacein, as assayed by the ethidium bromide/acridine orange assay (**figure 2c**), is at bay with a major role for violacein on TF1 cell survival. Thus, death of TF1 cells following application of violacein seems not to be mediated by one of the canonical PCD modalities. In apparent agreement, violacein induced neither increased expression of Beclin-1 nor massive formation of the autophagosome associated form of LC3-B (**figure 2d**), further excluding an important role of autophagic cell death in violacein-mediated cell death. Nevertheless, violacein treatment slightly increased the levels of cleaved PARP (**figure 2d**).

### Ultrastructural characterisation of violacein effects in TF1 cells reveals endoplasmic reticulum and Golgi linearization

**Figure 3:**
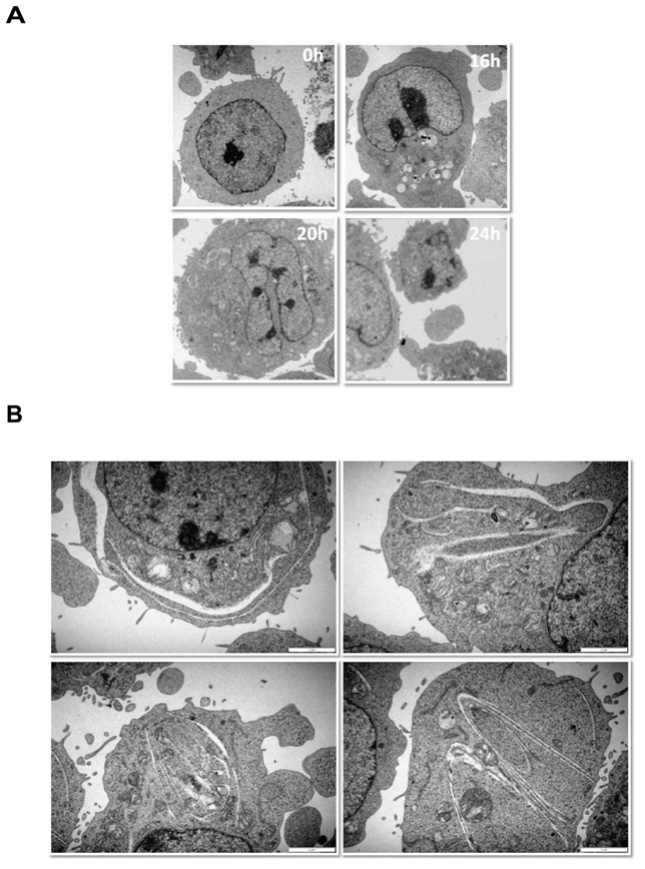
Induction of cell death by violacein is associated with an ultrastructurally unique programme of cellular demise. (A) Using transmission electron microscopy, the ultrastructural effects of violacein-induced cell death were characterised at different time points following application of the indole derivative. No apparent morphological evidence for induction of either autophagy, apoptosis, necroptosis or pyroptosis is obtained, as for instance the integrity of the mitochondria and plasma membrane is maintained until the final stages of cellular suicide and no increase in the number of autophagosomes is seen. Highly distinctive of violacein-induced cell death, however, is the endoplasmic reticulum and Golgi linearization and at later time points nuclear mottening. (B) High magnification examples of endoplasmic reticulum and Golgi linearization, characteristic for violacein effects 20 hrs following application of the chemopreventive indole.

To obtain further insight into the mechanisms underlying TF1 cell death following violacein treatment, the ultrastructure of these cells was studied using transmission electron microscopy (**figure 3a**). Plasma membrane integrity was maintained until almost the final stages of cellular suicide and no increase in the number of autophagosomes was observed. In addition, no apparent morphological evidence for induction of autophagic cell death (as the vesicles observed do not present double membranes which is characteristic of this type of cell death), apoptosis or necroptosis was observed. However, violacein-induced cell death was followed by endoplasmic reticulum and Golgi linearization (see high power magnification pictures in **figure 3b**), and ,at later time frame, the appearance of ‘horseshoe-shaped’ nuclei, initiating the last phase before cellular demise, becomes prominent.

### Violacein caused a dynamic reprogramming in TF1 cells kinases assessed by Western Blot

**Figure 4:**
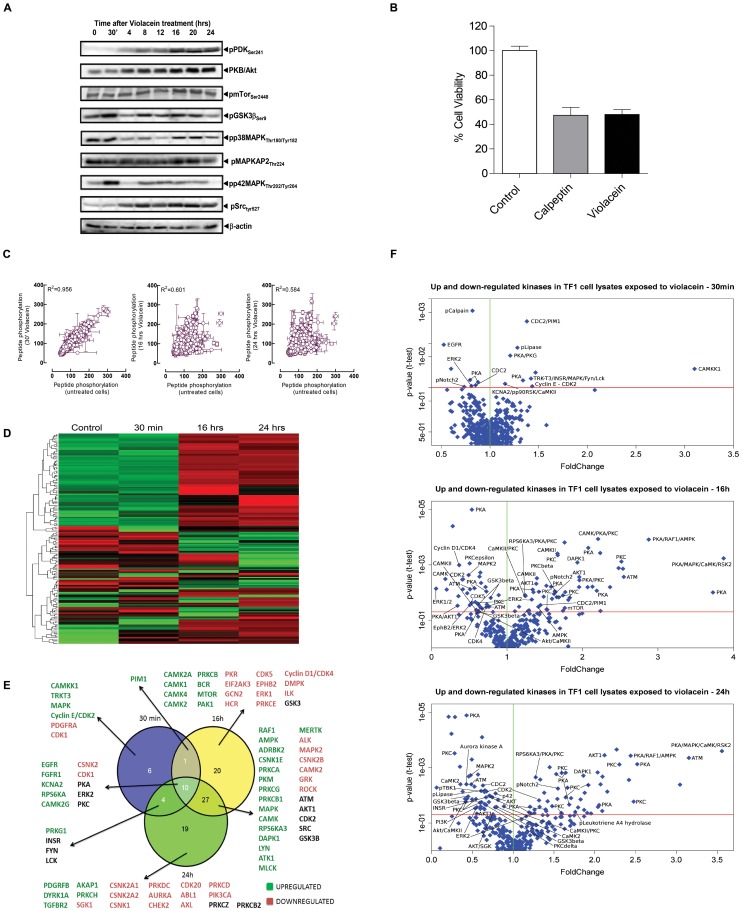
Induction of cell death by violacein is associated with a major rearrangement of cellular biochemistry. (A) The PKB-mediated survival cassette is not inhibited by 2 µM violacein treatment in TF1 cells. Although Western Blot analysis of signalling intermediate phosphorylation does not perfectly correlate with the kinase enzymatic activity, it is obvious that also phosphorylation status analysis does not provide evidence for diminished survival signalling following TF1 cell stimulation. Note that GSK3β activity is negatively regulated by its PKB-mediated phosphorylation (B) Viability of TF1 cells was evaluated in the presence of Calpeptin (50 µM) or after violacein (2 µM) treatment. (C) Plotting correlation between phosphorylation of specific peptide substrates shows that 30 minutes following violacein treatment only minor changes in the cellular kinome are observed. More long-term treatment, however, causes major remodelling of the kinome. The value in the graph gives the Pearson product. (D) Heatmap of significantly altered kinases in TF1 cells in response to violacein treatment. (E) Venn diagram depicting the distribution of phosphorylated spots at different time points (30 min is depicted in blue, 16 hrs in yellow and 24 hrs in green). (F) Comparison between statistically significant phosphorylated spots in TF1 cells treated with 2 µM violacein for 0 hrs with cells treated for 16 hrs and 24 hrs. This graph shows the correlation between fold change and p-values for the statistically significant phosphorylated spots at the different time points of treatment with violacein.

Due to the strong inhibitory effect of violacein on the TF1 cell proliferation rate, the modulation status of some kinases was examined by western blot. PDK and AKT presented higher phosphorylation levels at positive regulatory sites, which indicated that violacein promotes activation of both enzymes. Src kinase was less active, since the inhibitory site at tyrosine residue 527 was phosphorylated. Activation of AKT, an important pro-survival kinase, could explain the absence of a strong effect of violacein on apoptotic and autophagic markers. On the other hand, the negative modulation of Src kinase might be, at least in part, responsible for the antiproliferative action of this compound (**figure 4a**).

mTOR kinase was not significantly affected by violacein. In relation to GSK, MAPKAP2, p38 and p42/44 kinases, we observed a transient influence of violacein along the evaluated time frame (**figure 4a**). These results indicate that the violacein-induced cell death is potentially triggered by oxidative stress as MAP kinases in these circumstances present themselves as pro-cell death signals [22]. Indeed, this is in line with the structures observed by EM in which we clearly observed ER and Golgi linearization probably caused by violacein-induced stress. Also, the strong activation of death associated protein kinase, which has been associated with PCD related to interferon-γ withdrawal but is also known to inhibit apoptosis and whose role in PCD thus remains obscure [23], is an obvious candidate mediator in violacein action. Particularly considering that this kinase seems to facilitate the interplay of different cell death subroutines [24]. In addition, the calpain L1 large subunit is significantly less phosphorylated in violacein-treated cells, an event associated with deactivation of this cysteine protease [25]. Importantly, defects in calpain functionality are associated with a variety of deficiencies, including lethality, dystrophy, and tumorigenesis, suggesting that a calpain-like system could be important to induce programmed cell death in these cells [26]. Hence, we investigated the capacity of calpeptin (an inhibitor of calpain enzymatic activity [27]) to mimic violacein effects on cell death and indeed we observed that such inhibition is almost equipotent to violacein in inducing cell death in TF1 cells (**figure 4b**), indicating that the calpain system also may play a role in PCD induced by violacein.

### Violacein caused a dynamic reprogramming in TF1 cells kinases assessed by Kinome Profiling

Absence of *a priori* assumptions of the biochemical mechanisms that mediate the morphological effects of PCD induced by violacein prompted us to look for techniques that allow the generation of unbiased and comprehensive descriptions of cellular signalling. One such technique is kinome profiling using peptide arrays. We have used this methodology successfully to unravel the signalling mechanisms mediating, amongst others, chemoprevention by coxibs in colorectal cancer [28] or the non-genomic mechanisms employed by the glucocorticoid receptor [29] to limit white blood compartment expansion. For the present study, we generated kinome profiles using peptide arrays by incubating TF1 cell lysates obtained from cultures either untreated or subjected to 2 µM Violacein treatments for respectively 30 min, 16 h and 24 h. The arrays incorporated substantial amounts of radioactivity and the technical quality of the profiles was good as the average Pearson product moment obtained for the technical replicas ranged from 0.78 to 0.90 (**Table S1**). Application of violacein to cells caused an important and dynamic kinome reprogramming, which progresses over time (**figure 4c**). This is perhaps better visualized in **figure 4d** which shows a heat map built from the significantly altered kinases in TF1 cells lysates in response to violacein. Violacein-treated cells present a remarkably different profile in later time points (16 and 24 hrs) which are more alike compared to control or violacein treatment for 30 min (**figure 4d**). In **figure 4e** a Venn diagram depicting the distribution of up and downregulated kinases at different time points is shown. Densitometric values for all substrates and the statistical significance of the results obtained compared to untreated cells for all time points are given in **Table S2**. In **figure 4f** graphs presenting a correlation between fold change and p-value for each time point compared to control are presented. Analysis of these profiles, however, produces little evidence for an involvement of canonical PCD pathways in violacein-induced effects in TF1 cells. As it is also shown in **figure 4f**, the PKB/mTOR pathway, whose activation is associated with inhibition of both apoptotic and autophagic PCD modalities [30], is not significantly affected by violacein treatment.

Kinome profiling brought out some kinases which are differently modulated along the treatment of TF1 cells with violacein. Importantly, in agreement with viability and cell cycle analysis, some kinases that favour cell cycle progression and cell survival, such as CDK, Rock, Axl and AurkA, were negatively modulated by violacein, after 16 and 24 h. On the other hand, a huge set of kinases appeared more active in treated TF1 cells. Among those, some have not been reported as mediators of cell death or reticulum stress. Importantly, violacein modulated two kinases that have been linked with reticulum stress and cell death via apoptosis and autophagy. Violacein caused an expressive activation of PKA, as observed by the higher phosphorylation level of its substrate CREB1 observed on the peptide chip. In addition, we detected an increase of autophosphorylation of DAPK1 at its inhibitory site by violacein treatment.

## Discussion

Violacein and related indolic compounds attract attention because of their presumed chemopreventive action, originally discovered from epidemiological studies in the Amazon basin [31]. Although clearly highly biologically active [13], [32], the mechanism by which violacein might interfere with tumor cells remains partially unclear. Nevertheless, it has become evident that despite the absence of induction of cell death in untransformed cells or low *in vivo* toxicity of the compound in humans and experimental animals [32]–[33], it is strongly cytotoxic towards a number of transformed cell types. Specifically in relation to leukaemia, we have shown earlier that this pigment induces apoptosis of human chronic myeloid leukaemia cells (HL60) by intrinsic and extrinsic pathways. In order to provide more information about the potential antileukemic action of violacein, in the present study we examined the effect of violacein on a chemoresistant CD34^+^/c-Kit^+^/P-glycoprotein^+^/MRP1^+^ TF1 leukaemia progenitor cell line. Our findings revealed that violacein was able to bypass the natural resistance of TF1 cells, mainly through activating kinases that promote reticulum stress. Interestingly, some kinases that are involved in cell death via apoptosis and autophagy were inhibited after violacein treatment. The induction of PARP cleavage and the resulting breakdown of the cellular genome as well as the absence of trypan blue incorporation during violacein-induced cell death indicate that violacein acts through a specific cellular suicide program. All currently described forms of cell death seem to a certain extent to be inhibitory to other forms of PCD [4] and the form induced by violacein does not appear to be an exception.

Studies of cellular biochemistry, ultrastructural morphology and inhibitors of apoptosis, autophagy and pyroptosis indicate that violacein acts through a molecular mechanism distinct from these known manifestations of cellular death. In apparent agreement, detailed ultrastructural characterisation of violacein effects on cellular morphology revealed a unique cascade of events involving first endoplasmic reticulum and Golgi linearization and at later time points the nuclei assume a ‘horseshoe shape’. Biochemically, these morphological events were accompanied by highly distinctive effects on the kinome and concomitant downregulation of the Calpain cysteine protease system. Despite kinome profiling allowed us to elect a set of kinases that was differentially affected by violacein, in this section we will point out those that gave us support to explain the fate of TF1 cells towards this pigment.

Violacein effects are accompanied by strong activation of survival and anti-autophagic/anti-apoptotic signalling pathways through the AKT and PDK activation and inhibition of DAPK1, which counteracts both apoptosis as well as autophagic cell death. Activation of AKT signalling induced by violacein might be due to reticulum stress, as it has been previously reported that AKT driven signalling is enhanced under reticulum stress. Some reports pointed out that endoplasmic reticulum and Golgi apparatus can positively modulate both pro-survival (mainly related to protection responses) mechanisms as well as cell suicide when the stress stimuli threshold is exceeded [34]. DAPK1 has been reported to be an important mediator of apoptosis and autophagy [35], [36] and, since it was observed that violacein caused an expressive inhibition of this kinase, this fact can explain, at least in part, the absence of identification of typical markers of apoptosis or autophagy in TF1 cells treated with violacein.

The exact biochemical details by which violacein-induced cell death progresses evidently require further analysis, but it is interesting to see that inhibition of calpain mimics important aspects of the effect observed in response to violacein, suggesting that inhibition of calpain enzymatic activity may be an important factor in this process. In apparent agreement, genetic ablation of calpain enzymatic activity strongly decreases the propensity to tumorigenesis in experimental animals [37].

In general, the data presented here provide basis to explain the broad action of violacein as antitumoral agent. It is important to emphasize that violacein was able to induce death of resistant leukaemia cells, and kinome determination was a valuable strategy to select crucial kinases for this mechanism.

## Supporting Information

Table S1 Technical quality of kinome profiling. Cells were lysed and incubated on peptide arrays (1024 spots from which represent 974 bona fide kinase consensus substrates and 50 technical controls) in the presence of 33P-γ-ATP. Subsequently for each substrate phosphorylation was determined using a phosphoimager, yielding a dataset. Three datasets of each condition were obtained by parallel incubation of peptide array, yielding dataset 1 through 3 for each condition (technical replicates). The results depicted in the table represent the Pearson moment between these technical replicates and was always in excess of 0.78.
(DOC)

Table S2 Results of kinome profiling of TF1 cells exposed for 30 min, 16 hrs or 24 hrs to 2 µM violacein compared to control. Following violacein incubation, cells were lysed and incubated on peptide arrays (1024 spots from which represent 974 bona fide kinase consensus substrates and 50 technical controls) in the presence of 33P-γ-ATP. Subsequently for each substrate phosphorylation was determined using a phosphoimager, yielding a dataset. Tables show peptide motifs employed by the corresponding source protein from which the peptide motif was obtained, the average phosphorylation obtained from three datasets and its standard deviation. For those peptides of which the phosphorylation following violacein treatment was statistically significant different from control cultures, the P value is given as well.
(XLS)
