# Supplementary material for: Violacein Induces Death of Resistant Leukaemia Cells via Kinome Reprogramming, Endoplasmic Reticulum Stress and Golgi Apparatus Collapse
Source: PLoS One. 2012 Oct 11;7(10):e45362. doi: 10.1371/journal.pone.0045362 (PMC3469566; doi:10.1371/journal.pone.0045362)
Supplement: Table S1 — Technical quality of kinome profiling. Cells were lysed and incubated on peptide arrays (1024 spots from which represent 974 bona fide kinase consensus substrates and 50 technical controls) in the presence of 33P-γ-ATP. Subsequently for each substrate phosphorylation was determined using a phosphoimager, yielding a dataset. Three datasets of each condition were obtained by parallel incubation of peptide array, yielding dataset 1 through 3 for each condition (technical replicates). The results depicted in the table represent the Pearson moment between these technical replicates and was always in excess of 0.78. (DOC) [file pone.0045362.s001.doc]

**Table S1** | Technical quality of kinome profiling. Cells were lysed and incubated on peptide arrays (1024 spots from which represent 974 *bona fide* kinase consensus substrates and 50 technical controls) in the presence of ^33^P-γ-ATP. Subsequently for each substrate phosphorylation was determined using a phosphoimager, yielding a dataset. Three datasets of each condition were obtained by parallel incubation of peptide array, yielding dataset 1 through 3 for each condition (technical replicates). The results depicted in the table represent the Pearson moment between these technical replicates and was always in excess of 0.78

0,852

0,849

0,897

0,907

1*vs*3

0,785

0,803

0,901

0,872

2*vs*3

0,852

0,849

0,897

0,907

1*vs*2

**24 *hrs***

**Violacein**

**16 *hrs* Violacein**

**30’ Violacein**

**Untreated**

**Sets**
